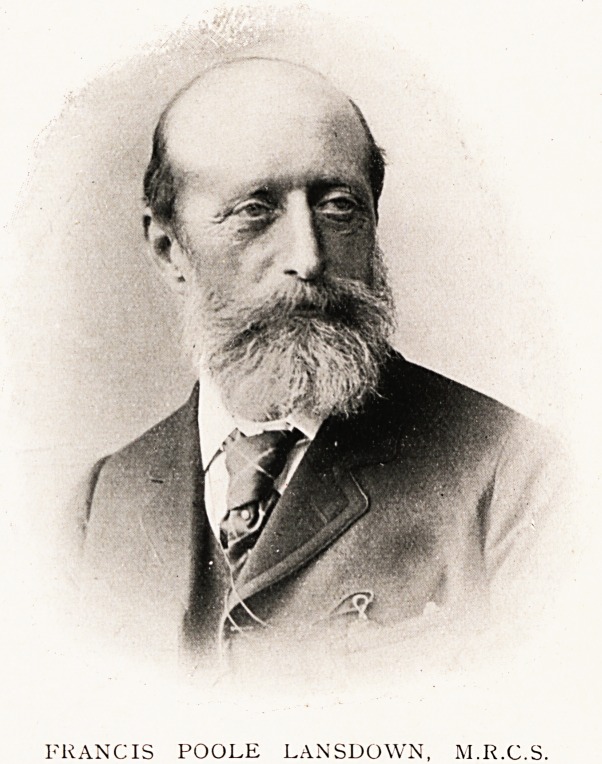# Francis Poole Lansdown, M.R.C.S., L.S.A.

**Published:** 1917-04

**Authors:** 


### FRANCIS POOLE LANSDOWN, M.R.C.S., L.S.A.

It is with a deep sense of the loss sustained by the medical life of the city that we record the death of Mr. Francis Poole Lansdown, Consulting Surgeon to the Bristol General Hospital, which occurred on the 12th February, 1917, after a short illness, at the ripe age of 83.

The name of Lansdown appears far back in the city annals. In the Great Red Book of Bristol is a record of the sale by Thomas Lansdown, of Bristol, of a house in Maryleport Street, 1542. Again, Will. Lansdowne (*sic*), chirurgeon, was admitted to the freedom of the city, for that he was apprenticed to John Webb, 1699; and further, Thomas Lansdown, barber-chirurgeon, was admitted to the freedom of the city, apprenticed to John King, 1689. A paternal aunt of Mr. Lansdown, born in 1800, was presented, on her marriage to Mr. Richard Blogh in 1836, with a framed pictorial acrostic of considerable local interest, executed by Stephen Jenner, brother of Edward Jenner, with whom the Lansdowns of that date were intimate.

Francis Poole Lansdown was born in 1833 in St. James's Barton. Upon his marriage he lived for some time in Lower College Green, where he secured a large private practice amongst the cathedral dignitaries. He subsequently moved to Park Street, then a residential street, and thence to Whiteladies Road, which he left only to retire to his beautiful Devonshire home at Lydford on Dartmoor. He had always taken great interest in his garden at Clifton, and met with considerable success as

FRANCIS POOLE LANSDOWN, M.R.C.S.

an amateur fruit-grower, a pursuit which continued to afford him pleasure and employment to the end.

The Lansdown family has been closely associated with the Bristol General Hospital since its opening in November, 1832. Some houses in Guinea Street first afforded a home to the Institution, until the growing needs of the district led to the erection of the Hospital on the present site. One of the first surgeons to be appointed was Mr. Joseph Goodall Lansdown, who had for his surgical colleagues G. D. Fripp, Henry Brigstocke, and John Grant Wilson. He was re-elected from time to time, and finally resigned in April, 1861, after twenty-nine years' continuous service, to be succeeded by his son, Francis Poole Lansdown, who in his turn gave thirty-two years' faithful service to the Institution. Three years later his son, Robert Guthrie Poole Lansdown, was elected Hon. Surgeon to the Hospital, a post he still holds. The office of Surgeon to the Bristol General Hospital has thus been held by three successive generations of the Lansdown family, and as Mr. F. P. Lansdown was placed on the honorary consulting staff of the Hospital on his retirement in 1893, there has been an uninterrupted period of more than eighty-five years during which the family has been officially connected with the Institution. The surgical family tree still blossoms, for a member of the fourth generation is now a student of Guy's Hospital, to carry on the tradition.

"As a surgeon," one of his colleagues writes, " he was a calm, careful, and skilful operator, taking no unnecessary risks ; his opinion in surgical cases was sound, and he kept himself well abreast of the surgery of the day." He held several public appointments, including the post of Surgeon to the Post Office and to the City School, which he retained until his retirement.

Mr. Lansdown found little time for sport, though horse-exercise was always grateful to him when time permitted. As with many busy men who work in the crowded life of cities, when on holidays bent he found " society, where none intrudes, by the deep sea, and music in its roar." As July came round each year he would take ship from Bristol or London Docks for a three weeks' cruise to Ireland or the coast of Scotland. Putting in, perchance, at Plymouth, a walk on the Hoe and a bathe were preparation for continuance of the voyage. Sometimes his family accompanied him, and beguiled his footsteps to Killarney Lakes or Bantry Bay, but he usually kept closely to the ship's track, and was punctilious in returning home, for inscrutable reasons, always in time for the August Bank Holiday.

Mr. Lansdown took small interest in medical politics, and seldom attended the Annual Meetings of the Association, but he was keenly interested in, and frequent attendant at, the meetings of the local medical societies, of which he was in turn President. He was also for many years a member of the Clifton

Medical Reading Society, and contributed to the medical journals some valuable notes on the treatment of Aneurism.

Mr. Lansdown will be always remembered socially for his genial and friendly nature, and for the help and encouragement he was ever ready to extend to the younger members of the profession. What better memorial could one hope for than this, that the remembrance in many hearts of kindly deeds may serve to keep his memory green.

## Figures and Tables

**Figure f1:**